# Mast Cell-Derived Proteases Induce Endothelial Permeability and Vascular Damage in Severe Fever with Thrombocytopenia Syndrome

**DOI:** 10.1128/spectrum.01294-22

**Published:** 2022-05-25

**Authors:** Yu-Na Wang, Yun-Fa Zhang, Xue-Fang Peng, Hong-Han Ge, Gang Wang, Heng Ding, Yue Li, Shuang Li, Ling-Yu Zhang, Jing-Tao Zhang, Hao Li, Xiao-Ai Zhang, Wei Liu

**Affiliations:** a State Key Laboratory of Pathogen and Biosecurity, Beijing Institute of Microbiology and Epidemiology, Beijing, Hebei, China; b College of Life Sciences, Fujian Agriculture and Forestry University, Fuzhou, Fujian, China; c School of Public Health, Anhui Medical University, Hefei, Anhui, China; Changchun Veterinary Research Institute

**Keywords:** SFTSV, chymase, mast cell, tryptase, vascular damage

## Abstract

Severe fever with thrombocytopenia syndrome (SFTS) is an emerging hemorrhagic fever acquired by tick bites. Whether mast cells (MCs), the body’s first line of defense against pathogens, might influence immunity or pathogenesis during SFTS virus (SFTSV) infection remained unknown. Here, we found that SFTSV can cause MC infection and degranulation, resulting in the release of the vasoactive mediators, chymase, and tryptase, which can directly act on endothelial cells, break the tight junctions of endothelial cells and threaten the integrity of the microvascular barrier, leading to microvascular hyperpermeability in human microvascular endothelial cells. Local activation of MCs (degranulation) and MC-specific proteases-facilitated endothelial damage were observed in mouse models. When MC-specific proteases were injected subcutaneously into the back skin of mice, signs of capillary leakage were observed in a dose-dependent manner. MC-specific proteases, chymase, and tryptase were tested in the serum collected at the acute phase of SFTS patients, with the higher level significantly correlated with fatal outcomes. By performing receiver operator characteristic curve (ROC) analysis, chymase was determined as a biomarker with the area under the curve value of 0.830 (95% CI = 0.745 to 0.915) for predicting fatal outcomes in SFTS. Our findings highlight the importance of MCs in SFTSV-induced disease progression and outcome. An emerging role for MCs in the clinical prognosis and blocking MC activation as a potential drug target during SFTSV infection was proposed.

**IMPORTANCE** We revealed a pathogenic role for MCs in response to SFTSV infection. The study also identifies potential biomarkers that could differentiate patients at risk of a fatal outcome for SFTS, as well as novel therapeutic targets for the clinical management of SFTS. These findings might shed light on an emerging role for MCs as a potential drug target during infection of other viral hemorrhagic fever diseases with similar host pathology as SFTS.

## INTRODUCTION

Severe fever with thrombocytopenia syndrome (SFTS) is an emerging tick-borne disease caused by a novel bunyavirus, SFTS virus (SFTSV, renamed as Dabie bandavirus in 2020) in the *Bandavirus* genus of the family *Phenuiviridae* (1). Since its first report in 2009, SFTS has been increasingly reported in the central, eastern, and northern regions of China and other Asian countries, including South Korea, Japan, Vietnam, and Myanmar (2–6). Due to its high fatality rate, SFTS had been listed in the top 10 diseases requiring prioritized research and intervention by WHO in 2017 (7). The clinical spectrum of SFTS ranges widely from asymptomatic carriage, influenza-like illnesses, and hemorrhagic fever to multiple organ failures, and even death in 12% to 50% of the hospitalized cases (1, 8). No licensed vaccines or specific antiviral therapies have been developed for the prevention and treatment of SFTS, thus raising concerns that SFTS will pose a severe public health threat.

SFTSV is mainly transmitted to humans by an infected tick vector (9). During the blood-feeding of infected ticks on humans, SFTSV is injected into the skin and bloodstream by ticks. As the port of entrance for SFTSV, the skin-resident cells that surround the bite location, such as immature Langerhans cells, epidermal dendritic cells (DCs), keratinocytes, and mast cells (MCs), which had been displayed to be infected in Dengue virus (DENV) (10), might be the targets for SFTSV infection. Many of these infected cells can then migrate from the site of infection to lymph nodes and other tissues, systemically spreading the infection (11). Among them, skin MCs are the most important sources of newly emerged virions and released host immune factors (12). MCs are granule-rich immune cells that are distributed throughout the body in areas where microorganisms typically reside, such as the skin, mucous membranes, and connective tissues. They are near blood vessels, with functional roles in a variety of inflammatory conditions (i.e., asthma and allergy) and innate protection against pathogens (13). Once encountered by pathogens, MCs are activated and respond in a two-stage manner: immediate degranulation within minutes, resulting in the release of presynthesized mediators, such as histamines, proteases, and other bioactive substances, and delayed secretion of secondary *de novo* synthesized mediators, mainly, including cytokines, chemokines, and growth factors that are released within hours of activation (14, 15). Chymase and tryptase, the major proteins stored and secreted by MCs, have been used as markers for MC activation and can induce the release of proinflammatory chemokines during viral infections (16). Thus, MC function not only as sentinels but also as modulators of innate and adaptive immune responses, ultimately influencing disease outcomes.

In recent years, many viral pathogens were demonstrated to activate MCs, such as DENV, influenza A virus (IAV), hantaviruses, Japanese encephalitis virus (JEV), and SARS-CoV-2 (17–21). However, the extent of MC degranulation in response to viral infection and any resulting functional implications are less clear. DENV was among the most intensively studied viruses for MCs involvement with the evidence encompassing MC activation and degranulation upon DENV infection, subsequent inflammatory stimuli release, vascular hyperpermeability, and leakage, which all correlate with disease severity (22–24). However, both beneficial and detrimental functions of MCs in dengue hemorrhagic fever (DHF) had been previously reported, yielding controversial conclusions as to its role in dengue pathogenesis (12). Owing to the transmission route via arthropod bites as well as clinical aspects involving hemorrhagic and plasma leakage that are common to DENV and SFTSV, we hypothesized that MCs might be involved in the immune response or pathogenesis during SFTSV infection.

## RESULTS

### Degranulation of MCs in response to SFTSV.

**FIG 1:**
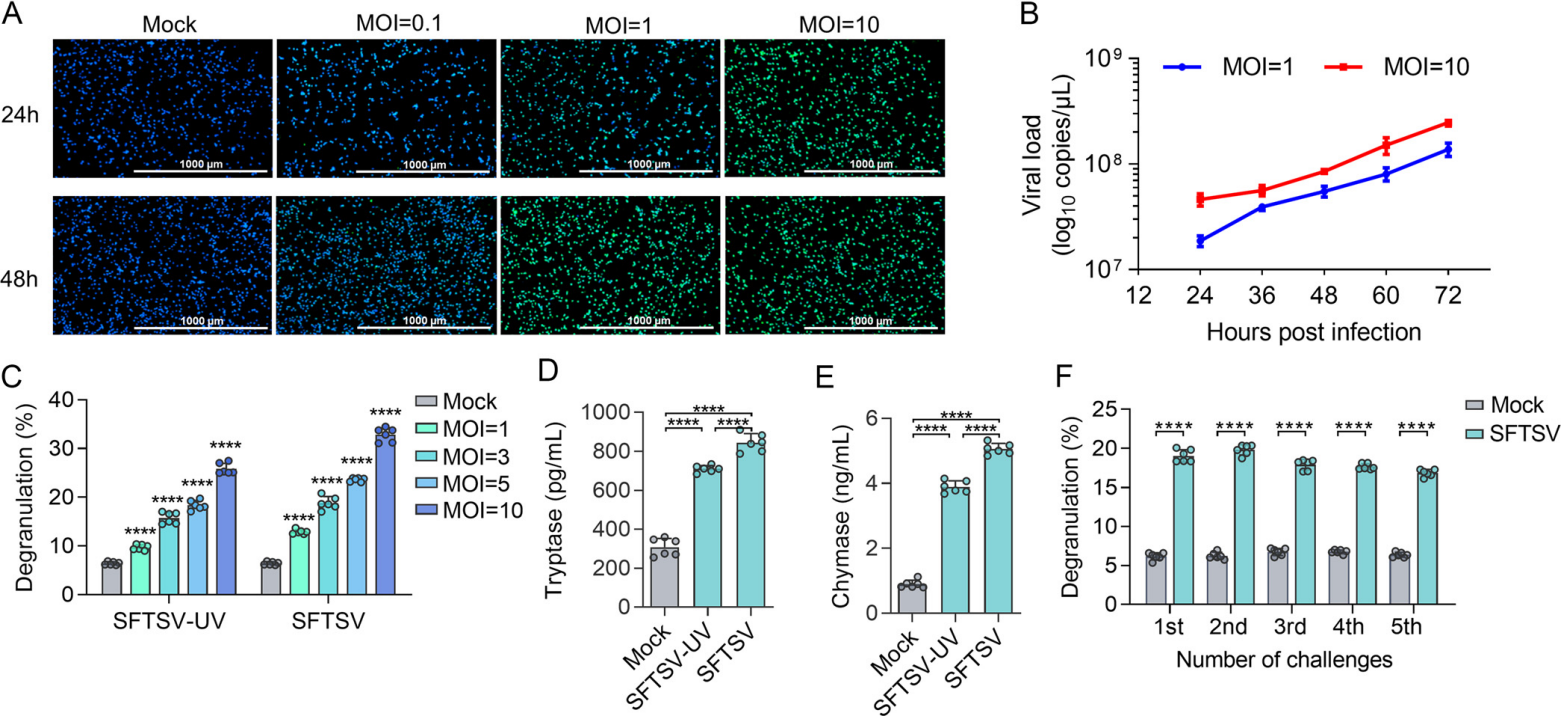
MCs degranulate in response to SFTSV. (A) Localized SFTSV-NP after MCs stimulated with SFTSV at a different time at various MOI. (B) The culture medium was harvested at the designated time points and the secretion of infected virions in MCs was measured. (C) Comparable MC degranulation to live and UV-inactivated SFTSV. The β-hexosaminidase assay was used to assess MC degranulation. (D) Comparable tryptase released by MC degranulation to live and UV-inactivated SFTSV (MOI = 1). (E) Comparable chymase released by MC degranulation to live and UV-inactivated SFTSV (MOI = 1). (F) Release of β-hexosaminidase in the supernatants of degranulated MCs that were challenged repeatedly with live SFTSV (MOI = 3) for 30 min, respectively. Untreated MCs were used as a control. In (C to E), multiple comparisons among the groups were made using the one-way ANOVA test followed by Bonferroni *post hoc* correction. In (F), the Student's *t* test was performed. Dots indicate exact value; histograms indicate mean and SD. Data from three independent experiments. ****, *P* < 0.0001.

To determine whether MCs are activated during SFTSV infection, we first examined the ability of SFTSV to induce degranulation in the human MC line (LAD2) with different multiplicity of infection (MOI) of strain HBMC16. The intracellular SFTSV virions in LAD2 cells increased in an MOI-dependent manner (Fig. 1A). A steady increase of infectious virions was observed in the cell supernatant within 72 h postinfection (hpi) for both MOI-1 and MOI-10 groups with the latter group showing persistently higher levels than the former (Fig. 1B). Exposure of MCs to both live and UV-inactivated SFTSV after 1 hpi showed significant degranulation of LAD2 cells with the level of β-hexosaminidase increased in relation to MOI (all *P* < 0.0001; Fig. 1C). The treatment of LAD2 cells with either live or UV-inactivated SFTSV for 1 h had significantly increased release of MC-derived proteases tryptase (all *P* < 0.0001; Fig. 1D) and chymase (all *P* < 0.0001; Fig. 1E). Next, live SFTSV (MOI = 3) was utilized to reactivate LAD2 cells repeatedly, from which a continuous release of β-hexosaminidase was observed (Fig. 1F). These results showed that SFTSV could effectively infect human MCs and replicate to induce MC degranulation, which appeared to be also in response to viral structural proteins because inactivated viruses prompted degranulation.

**FIG 2:**
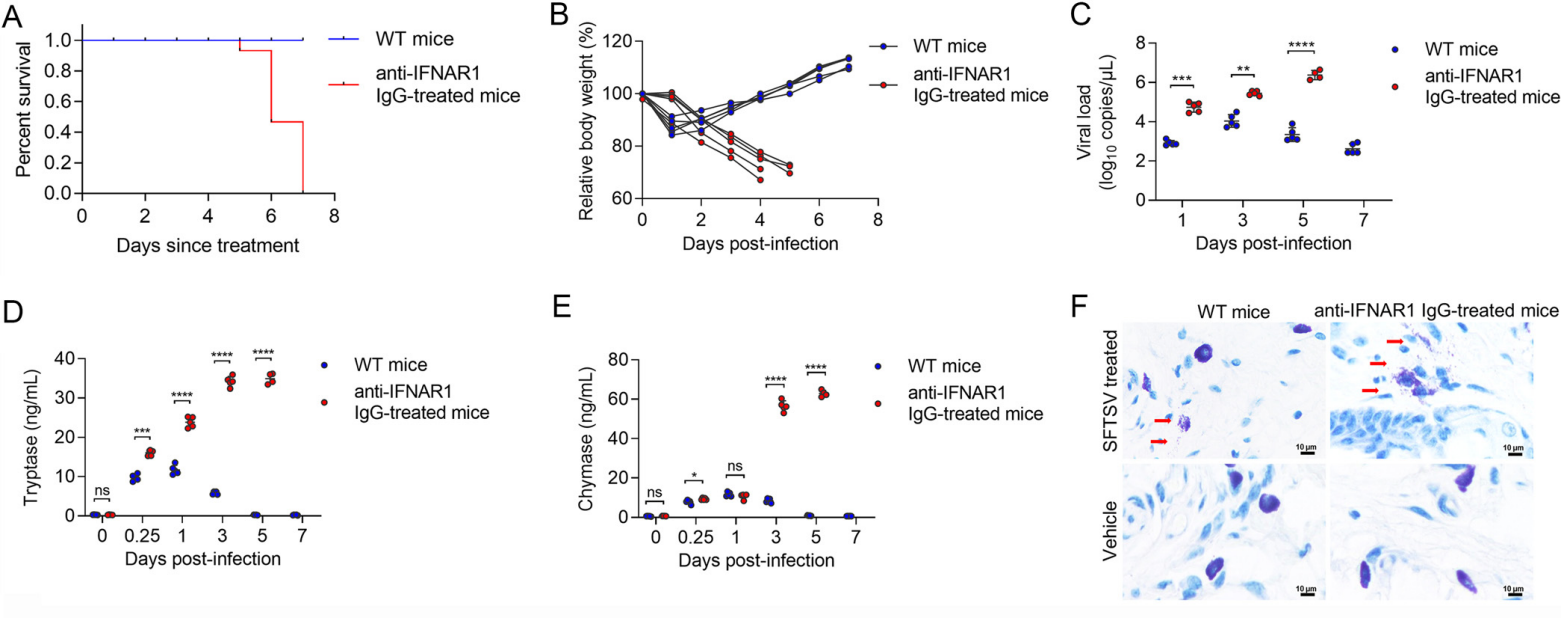
MC activation in response to SFTSV *in vivo*. (A) Survival curves of WT mice and anti-IFNAR1 IgG-treated mice infected with SFTSV. (B) Changes in body weight of WT mice and anti-IFNAR1 IgG-treated mice infected with SFTSV. (C) Changes in serum viral load of WT mice and anti-IFNAR1 IgG-treated mice infected with SFTSV after post-intraperitoneal infection. (D) Changes in serum tryptase of WT mice and anti-IFNAR1 IgG-treated mice infected with SFTSV after post-intraperitoneal infection. (E) Changes in serum chymase of WT mice and anti-IFNAR1 IgG-treated mice infected with SFTSV after post-intraperitoneal infection. (F) Images of WT and anti-IFNAR1 IgG-treated mouse footpad sections of control and SFTSV-injected footpads, as assessed by toluidine blue staining. The 2 × 10^5^ PFU of SFTSV were injected for WT mice and 3 × 10^3^ PFU SFTSV were injected for anti-IFNAR1 IgG-treated mice. Dots indicate exact value; histograms indicate mean and SD. Data from three independent experiments.

To corroborate whether MCs were able to degranulate in response to SFTSV *in vivo*, we examined serum levels of the MC-specific proteases tryptase and chymase in both WT mice (nonlethal SFTSV-infection model) and anti-IFNAR1 IgG-treated mice (lethal SFTSV-infection model) (Fig. 2A). In anti-IFNAR1 IgG-treated mice, lower body weights and higher serum viral loads were observed during the whole course of infection compared with WT mice (Fig. 2B and C). In WT mice, serum tryptase and chymase levels were significantly elevated at 6 hpi, peaking at 1 day after infection, and then gradually decreased to baseline levels at 5 to 7 days postinfection (Fig. 2D and E). In a differing manner, serum levels of tryptase and chymase in anti-IFNAR1 IgG-treated mice were significantly elevated and persistently increased until the dead-end (Fig. 2D and E). MC degranulation was further observed by injecting SFTSV into the footpads of WT mice and anti-IFNAR1 IgG-treated mice, respectively. At 6 h after treatment, footpads were sectioned and stained with the MC-specific stain, toluidine blue. MC degranulation was shown from SFTSV-injected footpad tissue, which was visualized by large amounts of extracellular granules in both WT mice and anti-IFNAR1 IgG-treated mice while not observed in the control-injected tissue (Fig. 2F). Together, these data suggested that activation of MCs could also occur *in vivo*.

### MC-derived products promoted endothelial permeability and broke tight junctions.

**FIG 3:**
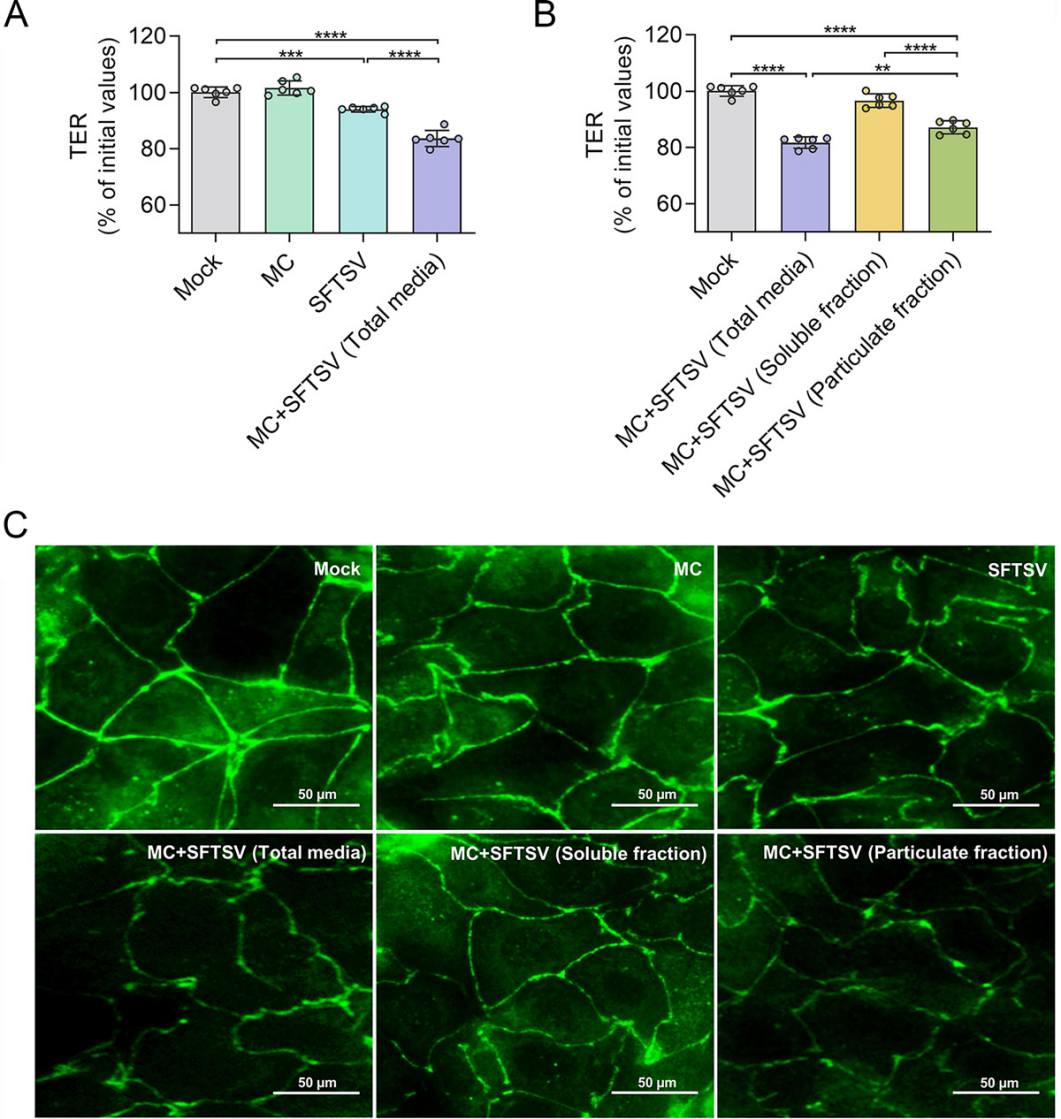
MC-derived particulate fraction promoted endothelial permeability and broke tight junctions. (A) MC, SFTSV, or SFTSV-induced MC total medium were transferred onto HUVEC dense monolayers, and then the TER changes of HUVEC monolayer cells were measured 24 h after treatment. For the vehicle control group, the untreated HUVEC cells were used. (B) SFTSV-induced MC total medium was transferred onto HUVEC monolayers or separated into soluble and particulate fractions, followed by transfer onto HUVEC monolayers. For the vehicle control group, the untreated HUVEC cells were used. TER changes in the HUVEC monolayer were measured 24 h after treatment. (C) HUVEC cells were treated with MC, SFTSV, and SFTSV-induced MC total media, soluble and particulate fractions from a total medium for 24 h followed by fixation and staining against tight junctions (ZO-1, green). Scale bars: 50 μm. For (A to C), MCs were stimulated by SFTSV with a dose of MOI = 1 for 1 hpi. In (A and B), multiple comparisons among the groups were made using the one-way ANOVA test followed by Bonferroni *post hoc* correction. Dots indicate exact value; histograms indicate mean and SD. Data from three independent experiments. **, *P* < 0.01; ***, *P* < 0.001; ****, *P* < 0.0001. TER, transendothelial electric resistance; hpi, hours postinfection.

To investigate whether the biological active medium derived from MCs could promote vascular injury during SFTSV infection, we determined the endothelial integrity by measuring transendothelial electric resistance (TER). Significantly decreased TER was observed in monolayers treated with SFTSV alone (*P* < 0.001), with an even greater reduction presented in the group treated with SFTSV-induced MC total medium (*P* < 0.0001) (Fig. 3A). Human umbilical vein endothelial cells (HUVEC) monolayers treated with the particulate fraction of SFTSV-induced MC total medium demonstrated significantly reduced TER compared with the soluble fraction treatment group (*P* < 0.0001) (Fig. 3B). No significant difference in TER was observed for the soluble fraction treatment group compared with the untreated control.

A previous study showed that both tryptase and chymase were able to break tight junctions between endothelial cells, causing increases in endothelial permeability during DENV infection (22). Here, we questioned whether disruption of tight junctions will also lead to the increase in endothelial permeability induced by SFTSV-elicited MC products. To address this, the HUVEC monolayer was treated with SFTSV-induced MC total medium, and a soluble and particulate fraction of SFTSV-induced MC total media, respectively, followed by staining for tight junction protein ZO-1. The microscopy images show uniform continuous staining of ZO-1 in the control group, in contrast to slight damage of tight junctions observed in HUVEC monolayers treated with a soluble fraction or SFTSV alone. Consistent with TER data presented in Fig. 3B, remarkable damage to tight junctions was observed in HUVEC monolayers treated with SFTSV-induced MC total medium or particulate fraction (Fig. 3C). These results indicated that endothelial damage might be induced by MC-derived granule-related components post-SFTSV infection.

### MC-specific proteases induced decreased expression of adhesion molecule CD31 on the vascular endothelial surface.

**FIG 4:**
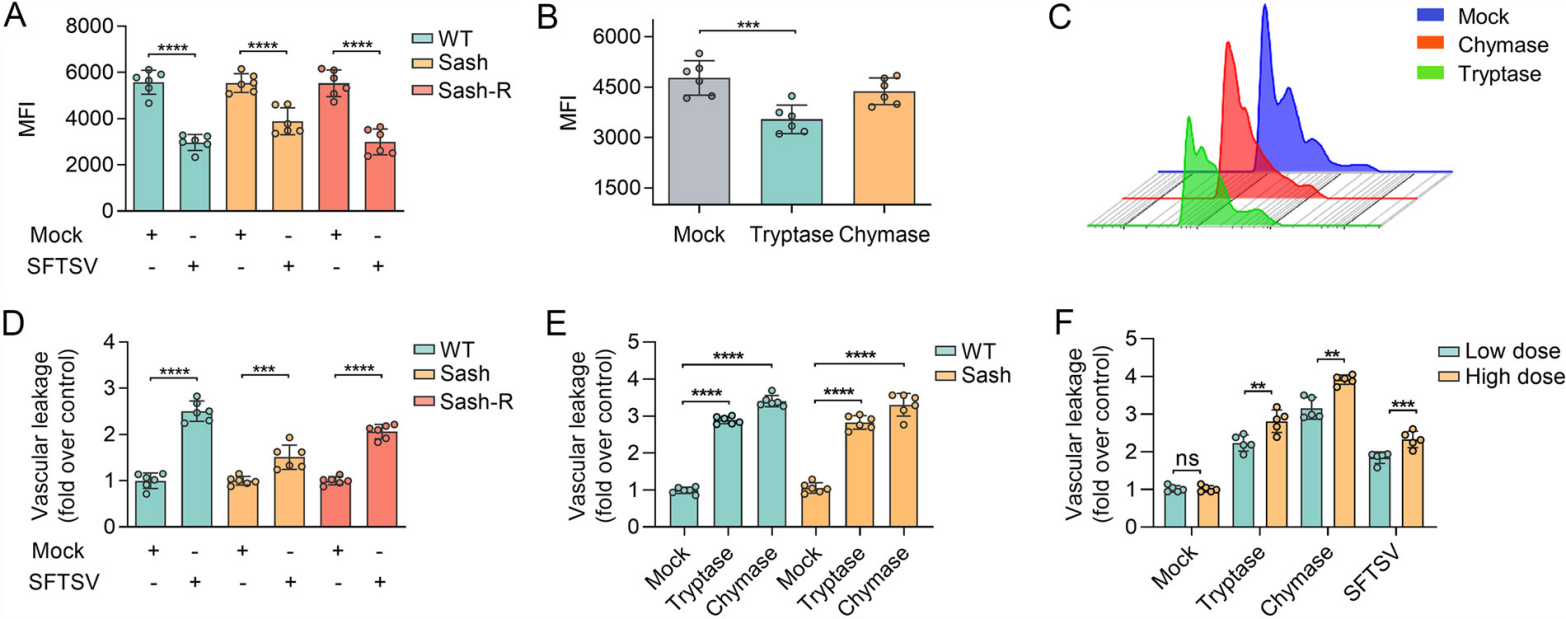
MC-derived specific proteases break tight junctions to disrupt endothelial cell contact sites, increasing vascular permeability. For (A to C), levels of CD31 on the surface of endothelial cells were measured by flow cytometry. (A) Comparison of MFI of CD31 staining in footpads of WT, Sash, and Sash-R mice at 6 h after injection of 1 × 10^6^ PFU SFTSV (*n* = 6 for each group). (B) Comparison of MFI of CD31 staining in WT mouse footpads after injection of 100 ng tryptase or chymase or an equal volume of normal saline control (*n* = 6 for each group). (C) CD45^−^, CD31^+^ cells showed reduced levels of staining after injection of chymase (representative histogram plots). For (D to F), examination of vascular leakage in mouse models by detecting extravasated Evans blue. WT, Sash, and Sash-R mice were intradermally injected with SFTSV, tryptase, chymase, or saline vehicle control for 3 hpi. Evans blue dye (2%, 250 μL) was intravenously injected into the mice, which were sacrificed 1 hpi (*n* = 6 for each group). (D) Evans Blue dye extravasation of back skin in WT, Sash, and Sash-R mice after intradermal injection of 1 × 10^6^ PFU SFTSV (*n* = 6 for each group). (E) Evans Blue dye extravasation of back skin in WT and Sash mice after intradermal injection of 100 ng tryptase or chymase or an equal volume of normal saline control (*n* = 6 for each group). (F) Evans Blue dye extravasation in WT mice after intradermal injection of 50 ng (low dose) and 100 ng (high dose) of tryptase or chymase, 1 × 10^5^ (low dose) and 1 × 10^6^ (high dose) PFU of SFTSV and an equal volume of saline (*n* = 5 for each group). Dots indicate exact value; histograms indicate mean and SD. The Student's *t* test (A and D) and the one-way ANOVA test (B, E, and F) were performed. Data from three independent experiments. ***, *P* < 0.001; ****, *P* < 0.0001; hpi, hours postinfection.

To further understand how endothelial cell damage is affected by MC-derived granular components *in vivo*, we detected the expression level of cell adhesion molecule CD31 (also known as PECAM-1, a functional component of the tight junction from endothelial cells that provides key evidence of endothelial cell damage) on the surface of vascular endothelial cells. SFTSV was injected into the footpads of MC-deficient (Sash) mice, MC-reconstituted Sash mice (Sash-R), and WT mice, from which tissue samples of the footpad were collected and digested into single-cell suspension after 6 hpi. Significantly decreased expression of CD31 was observed on endothelial cells from all three mouse models post SFTSV infection, with an even greater reduction presented in WT and Sash-R mice (Fig. 4A). MC-specific proteases, tryptase, and chymase were further injected into mouse footpads of WT mice, based on which a significant reduction in the surface expression of CD31 was observed in tryptase-treated mice compared to the control group. Yet, chymase-treated mice experienced a weaker decrease in CD31 expression compared with the tryptase-treated mice (Fig. 4B and C). These data suggest MC-derived proteases might be involved in endothelial cell damage by reducing the expression of CD31 on the vascular endothelial surface.

### Effect of MC-specific proteases on microvascular barrier destruction *in vivo*.

To investigate whether MC-derived products could damage the barrier integrity of local microvessels, microvascular permeability was evaluated in the three mouse models. Intradermal injections of SFTSV into dorsal skin induced a greater increase in the physiological aspects of Evans blue leakage in the WT and Sash-R mice compared with Sash mice (Fig. 4D), suggesting that MCs can effectively alter the microvascular barrier integrity *in vivo*. By subcutaneously injecting tryptase or chymase into the dorsal skin of WT and Sash mice, we observed microvascular hyperpermeability that was induced in a dose-dependent manner compared with the control group (Fig. 4E and F). Together, these findings revealed that SFTSV-induced MC-specific proteases could affect microvascular permeability and promote significant vascular leakage.

### MC-derived chymase and tryptase were correlated with SFTS fatal outcomes.

**FIG 5:**
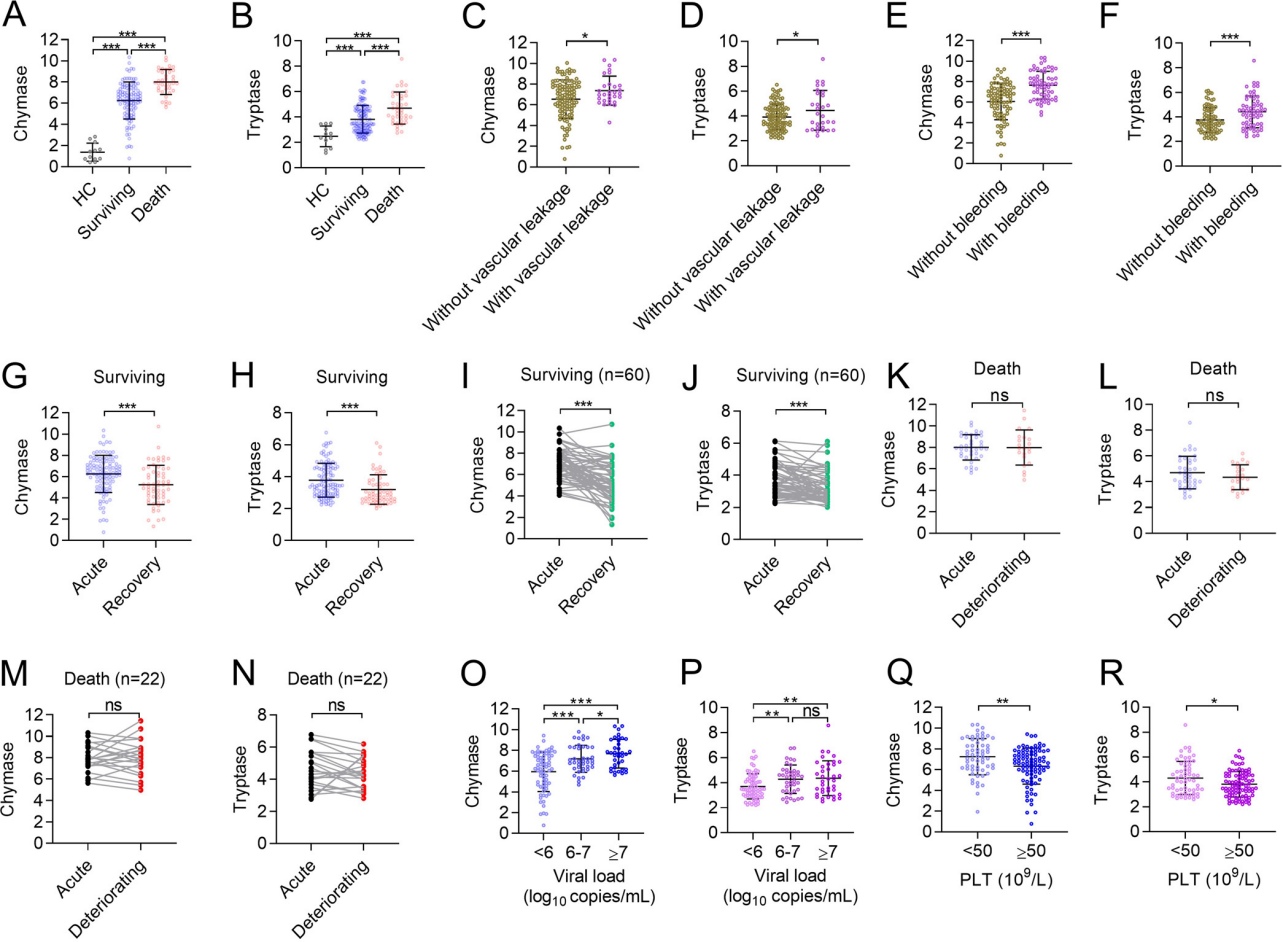
MCs specific chymase and tryptase in disease progression of SFTS patients. Levels of chymase (A) and tryptase (B) in serum samples from SFTS patients at the acute phase of illness (surviving, *n* = 108; death, *n* = 39) and healthy controls (*n* = 12). Levels of chymase (C) and tryptase (D) in serum samples from SFTS patients with (*n* = 30) or without (*n* = 117) vascular leakage. Levels of chymase (E) and tryptase (F) in serum samples from SFTS patients with (*n* = 61) or without (*n* = 86) bleeding. Levels of chymase (G and I) and tryptase (H and J) in serum samples from survived SFTS patients at acute (*n* = 108) and recovery (*n* = 60) phase of illness. Levels of chymase (K and M) and tryptase (L and N) in serum samples from deceased SFTS patients at acute (*n* = 39) and deteriorating (*n* = 22) phase of illness. Levels of chymase (O) and tryptase (P) in serum samples from SFTS patients stratified by viral load (*n* = 70 for <10^6^ copies/mL; *n* = 40 for 10^6^ to 10^7^ copies/mL; *n* = 37 for ≥10^7^ copies/mL). Levels of chymase (Q) and tryptase (R) in serum samples from SFTS patients were stratified by PLT count (*n* = 61 for <50 × 10^9^/L; *n* = 86 for ≥50 × 10^9^/liter). Dots indicate exact value; horizontal lines indicate mean and SD. The Student's *t* test (C to N and Q to R) and the one-way ANOVA test (A, B, O, and P) were performed. *, *P* < 0.05; **, *P* < 0.01; ***, *P* < 0.001; ****, *P* < 0.0001.

To explore the clinical significance of MC activation, we carried out a retrospective clinical study to evaluate the role of MC-specific proteases (chymase and tryptase) in the prognosis of SFTS. Totally 147 patients (61.9% female; median (IQR) of age: 66 (60, 71) years old) with laboratory-confirmed SFTS were tested for the level of chymase and tryptase in acute-phase serum samples and compared with those tested from 12 healthy controls (50% female; median (IQR) of age: 65 (65, 70) years old) (Table S1). Significantly increased levels of chymase and tryptase were observed in SFTSV-infected patients, and to an even higher level in 39 deceased patients than in 108 surviving ones (Fig. 5A and B). SFTS patients with clinical complications of bleeding (*n* = 61) or vascular leakage (*n* = 30) were also determined to express significantly higher levels of chymase and tryptase compared with those without such symptoms (Fig. 5C to F). The test on consecutively collected samples during the late hospitalization revealed different patterns for chymase and tryptase between deceased and surviving patients, which was significantly reduced among the survivors (Fig. 5G to J) while maintained at similarly high levels as in the acute phase among the deceased patients (Fig. 5K to N). Viral load and platelet (PLT), as important predictors for poor prognosis of SFTS (25, 26), were also evaluated, with significantly higher levels of chymase and tryptase observed in those with a high viral load than low viral load (Fig. 5O and P), and in those with PLT counts <50 × 10^9^/L than ≥50 × 10^9^/L (*P* < 0.01 for chymase, *P* < 0.05 for tryptase) (Fig. 5Q and R). Nine adhesion molecules related to vascular endothelial dysfunction were tested for their correlation with MC-specific proteases. It was determined that four of them, including VEGF-A, SAA-1, VCAM-1, and ICAM-1 were significantly increased in the patients with either higher chymase or tryptase levels than the median level calculated from a different number of patients (chymase <6.82 ng/mL, *n* = 43; chymase ≥6.82 ng/mL, *n* = 46; tryptase <3.80 ng/mL, *n* = 52; tryptase ≥3.80 ng/mL, *n* = 37), while two of them, including PECAM-1 and P-selectin were significantly decreased (Fig. S1). The multivariate logistic regression further confirmed a significant association between increased risk of death and high chymase level (≥6.82 ng/mL) in the acute phase after adjusting the effect from age, sex, and delay from disease onset to hospital admission (adjusted OR 3.01, 95% CI = 1.35 to 7.11, *P* < 0.01) (Table S2). The findings indicated that MC-specific proteases may be induced to a significantly high level that was related to the clinical outcome.

**FIG 6:**
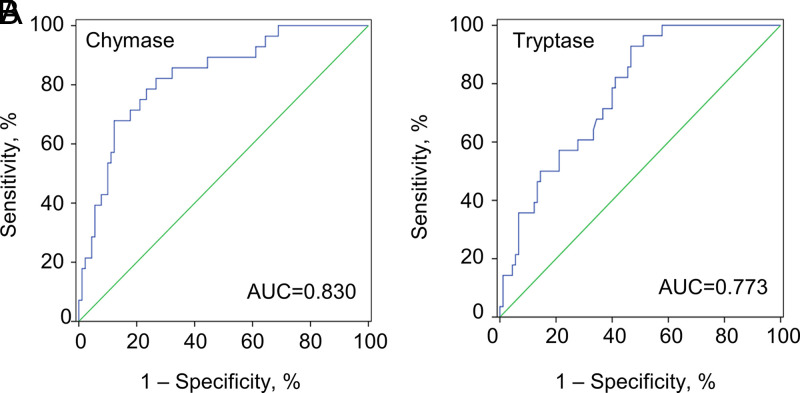
ROC analysis of MC-specific proteases in disease progression of SFTS patients. ROC analysis of chymase (A) and tryptase (B) levels in predicting the fatal outcome of SFTS patients admitted to hospital within 7 days of symptom onset (*n* = 118).

The predictivity of MC-specific proteases in the development of fatal outcomes in SFTS patients admitted to the hospital within 7 days of symptom onset was evaluated by the ROC analysis. The usage of chymase in model construction attained good prediction capacity, with the area under the curve (AUC) value of 0.830 (95% CI, 0.745 to 0.915), which was significantly higher than that of tryptase (0.773, 95% CI, 0.685 to 0.861). Analysis of SFTS patients revealed a sensitivity of 0.82 and a specificity of 0.74 when a chymase level of 7.16 ng/mL was used as a cutoff for a fatal outcome, and a sensitivity of 0.93 and a specificity of 0.53 when a tryptase level of 3.51 ng/mL was used as a cutoff for the fatal outcome (Fig. 6A and B). The findings showed that serum chymase level could act as an early biomarker to achieve a good prediction of fatal outcomes.

## DISCUSSION

Recent studies have shown the adverse role of MC in promoting the disease progression triggered by multiple viruses, e.g., involving in SARS-CoV-2-initiated lung inflammation and injury, promoting JEV infection in the central nervous system, and contributing to hantavirus infection-related vascular leakage syndrome (19–21). Our findings for the first time, highlight the importance of MCs in SFTSV-induced pathogenesis. We demonstrated that both live and inactivated SFTSV can trigger the activation of MCs to release biologically active mediators chymase and tryptase, which can increase endothelial cell permeability. Mechanistically, chymase and tryptase induced increased vascular permeability and leakage by reducing the expression of ZO-1 on endothelial cells, resulting in the loss of tight junctions. Importantly, the effect of chymase and tryptase by both *in vitro* and *in vivo* experiments was demonstrated in human microvascular endothelial cells and animal models. Further clinical evaluation of SFTS patients demonstrated the utility of chymase as a prognostic biomarker for SFTS.

The release of potent immune mediators prestored in the granules is the main mechanism by which MCs play roles in diverse pathophysiological processes. Some of these mediators are released immediately after MC activation and can act directly on vascular endothelium, including proteases (e.g., chymase and tryptase), histamine, and heparin (23). However, MC degranulation may not occur during infection with all viral pathogens, that might depend on the type of pathogen stimulus, location of pathogen recognition, and sensitization state of the responding MCs (27–29). Here, we showed the release of chymase and tryptase in the early inoculation of SFTSV within 1 h on human MCs, which was determined as an immediate response to SFTSV infection. The MC activation, proximately represented by the expression level of chymase and tryptase, was restored to a normal level at the late clinical phase of the survivors, corresponding to the clinical improvement of the SFTS patients. In contrast, the chymase and tryptase levels were significantly elevated and never restored in the fatal SFTS patients, indicating that when an individual is bitten by an SFTSV-carrying tick or infected with the pathogen through blood transmission, MCs may continue to be activated throughout the viremia with detrimental effect. Recently MCs have been increasingly recognized for their negative regulatory or feedback responses. Therefore, the failure of containment of MC activation might contribute to the detrimental outcome (30). This early initiation and the wide difference between survived and fatal SFTS patients have made chymase and tryptase potential prognostic factors in discriminating fatal cases at the early phase of SFTSV infection. Indeed, by applying AUC, we determined that chymase has good power in discriminating fatal cases.

Underlying the correlation between MC activation and SFTS fatal outcome, we also uncovered the potential mechanism by which SFTSV can induce vascular pathology through MC-derived proteases chymase and tryptase, which had been demonstrated for DENV pathology (23). DHF patients showed higher serum levels of chymase than those with dengue fever (23), highlighting the role of activation of MCs, which in turn causes significant peripheral vascular leakage and excessive inflammatory response and bleeding (24). Resembling DENV infection, it is believed that vascular leakage and bleeding are among the critical pathological processes in SFTS-related death (31). During this process, both direct endothelial infection by SFTSV and dysregulated immune response contribute to vascular endothelium dysfunction and barrier integrity deficiency (32). Here, we confirmed the role of MC-specific proteases chymase and tryptase in promoting SFTSV-induced vascular injury. *In vivo* experiments demonstrated that chymase and tryptase can induce vascular leakage in a dose-dependent manner in mouse models. These results were corroborated by the clinical data, showing elevation of both chymase and tryptase in SFTS patients, which were increased to an even higher level in those presenting vascular leakage and bleeding, moreover, evidenced by dysregulation of adhesion molecules that were indicative of endothelial dysfunction observed in those with higher chymase and tryptase release.

In conclusion, our results improve the knowledge of the pathogenic mechanism that could lay the groundwork for further evaluation of effective SFTSV therapy via blocking MC activation. Because medications that block MCs or their mediators have long been used to treat allergy and asthma (33), the use of protease inhibition for limiting the adverse effects of SFTS warranted further investigation. Our findings also point to the role of chymase as a useful biomarker for predicting fatal SFTS. Further studies are required to investigate the sensitivity, specificity, and predictive value of the chymase at various time points, and among subgroups of patients.

## MATERIALS AND METHODS

### Cell lines, virus strain, and culture conditions.

Human umbilical vein endothelial cells (HUVEC, RRID: CVCL_2959) were obtained from American Type Culture Collection (ATCC, USA) and maintained in Endothelial Cells Medium (ScienCell, USA) with 10% fetal bovine serum (FBS, Gibco, USA). Human MC line LAD2 was obtained from Bluefbio Biology Technology Development Co., Ltd. (Shanghai, China) and maintained in RPMI 1640 medium (Gibco, USA) containing 10% FBS. SFTSV strain HBMC16, which was isolated from the serum of an SFTS patient (34), was propagated in Vero cells and used for *in vitro* and *in vivo* experiments. Viral titer was determined by immunological focus assay on Vero cells. Inactivated SFTSV was obtained by exposing it to ultraviolet (UV) light for 3 h, and the inactivation was confirmed by an immunological focus on Vero cells.

### Assessment of MCs degranulation.

A β-hexosaminidase assay was used to assess MC degranulation *in vitro*. LAD2 cells were inoculated with SFTSV at a multiplicity of infection (MOI) of 1, 3, 5, or 10 for 1 h. LAD2 suspension was then collected and separated into supernatant and cell pellet by centrifugation, and the cell pellet was lysed by Triton X-100. β-hexosaminidase levels were measured in both supernatant and cell lysate (detailed in Text S1).

### Determination of endothelial integrity.

The endothelial integrity was determined by a transwell assay. HUVEC cells were cultured on the upper chamber of transwell inserts (Corning, USA) to form a tight endothelial monolayer, followed by treatment of the monolayers with SFTSV-induced MC total media. The soluble fraction (comprise *de novo* synthesized products and part of prestorage medium) and the particulate fraction (comprised insoluble prestorage medium, such as heparin, as well as proteases) of SFTSV-induced MC total medium were further separated by centrifugation (35). The soluble fraction and particulate fraction of SFTSV-induced MC total medium were then treated with HUVEC monolayer, respectively. Monolayer permeability was determined by acquiring transendothelial electric resistance (TER) measurement using the Millipore Millicell-ERS (Electrical Resistance System) (36) (detailed in Text S1).

### Immunofluorescence assay.

HUVEC cells were grown on coverslips (Solarbio, China) inside 24-well plates to form a tight endothelial monolayer, and then incubated with SFTSV-induced MC total media, and a soluble and particulate fraction of total media, respectively. LAD2 cells were inoculated with SFTSV at an MOI of 0.1, 1, or 10 for 24 and 48 h. The treated LAD2 cells or HUVEC monolayers were fixed with paraformaldehyde, permeabilized with Triton-100, and blocked using BSA blocking buffer. Cells were stained with mouse polyclonal antibody against SFTSV nucleoprotein (NP) protein or rabbit polyclonal antibody against ZO-1 (RRID: AB_2533456, Invitrogen, USA), followed by the addition of the secondary antibody, and counterstained with ProLongTM Gold Antifade reagent containing DAPI (Invitrogen). Images were obtained with the Leica DMi8 system (Leica, German).

### Quantitation of viral load.

Total RNA was extracted from the supernatant of SFTSV treated LAD2 cells and serum of mouse models after SFTSV infection, using the QIAamp MinElute Virus Spin kit (Qiagen, German). The quantitative real-time PCR (qRT-PCR) was performed with the One-step Primer Script RT-PCR kit (TaKaRa, Japan) in a LightCycler 480 (Roche). Virus loads were determined as previously described and expressed as copy/mL (37).

### Mouse experiments.

Five-week-old female wide-type (WT) mice on a C57BL/6 background were purchased from Charles River Laboratories (Beijing, China). WT mice were treated with anti-IFNAR1 (interferon-alpha/beta receptor 1) immunoglobulin G (IgG) (Bio X Cell, USA) (38) and then challenged intraperitoneally with SFTSV. Serum was collected at hour 6, day 1, day 3, day 5, and day 7 after injection, and chymase and tryptase levels were measured using a commercially available ELISA kit (Cloud-Clone Corp., China). Toluidine blue staining was carried out in mouse footpads by staining with a 0.1% solution of toluidine blue after injection of SFTSV or normal saline.

MC-deficient mice (Kit*^W-sh^*/*^W-sh^*; “Sash”) were purchased from Jackson Laboratory. For reconstitution of bone marrow-derived MCs in Sash mice to generate reconstituted Sash (Sash-R) mice, bone marrow cells were flushed from WT femurs and cultured in RPMI medium with 10% FBS, penicillin-streptomycin, recombinant murine IL-3, and recombinant murine stem cell factor (PeproTech, USA) for a period at which time bone marrow-derived mast cells (BMMCs) with more than 98% purity were used by toluidine blue staining. Sash mice received BMMCs via tail vein to reconstitute MCs and were allowed to mature.

Flow cytometry was used to assess endothelial cells stimulated with chymase or tryptase or SFTSV by subcutaneous injection in the mouse footpads of WT mice, Sash mice, and Sash-R mice. Next, mouse footpads were digested into single-cell suspensions using collagenase (Merk, USA) and stained with antibodies. For vascular permeability assay, mice were injected intradermally with SFTSV, chymase, tryptase, or normal saline into the shaved back skin followed by an intravenous injection of Evans Blue (Sigma). The dorsal skin (8 mm) containing the site of injection was removed. The Evans blue dye was extracted by incubation with formamide (Sigma), and its content was quantified at 610 nm by spectrophotometry. The animal experiment was approved by the Institutional Animal Care and Use Committee and was performed in accordance with the National Institutes of Health guidelines under protocols.

### Detection of MC proteases, cytokines, and adhesion molecules in SFTS patients.

Laboratory-confirmed SFTS patients with positive SFTSV RNA detection by qRT-PCR were recruited from the PLA 990 hospital, Xinyang in Henan province. Sera were collected at the acute and late phases of illness. Data about demography, clinical features, laboratory data, and outcome, were retrospectively collected. Healthy individuals that were comparable in age and sex were recruited as the control group. Informed consent was obtained from all individuals. The study was approved by the Ethical Committee of the Beijing Institute of Microbiology and Epidemiology.

Levels of chymase and tryptase in the serum were tested by commercial ELISA kits (Abbexa, UK and Cloud-Clone Corp., China). A series of cytokines and adhesion molecules related to vascular endothelial dysfunction, including platelet endothelial cell adhesion molecular (PECAM-1), vascular endothelial growth factor A (VEGF-A), serum amyloid antigen 1 (SAA-1), vascular cell adhesion molecular 1 (VCAM-1), intercellular adhesion molecular 1 (ICAM-1), P-selectin, E-selectin, tissue plasminogen activator (tPA) and plasminogen activator inhibitor 1 (PAI-1) were tested in the serum by the ProcartaPlex multiplex immunoassays panels (Affymetrix, USA).

### Statistical analysis.

Continuous variables were summarized as mean and standard deviation (SD) or as the median and interquartile range (IQR). Categorical variables were summarized as frequencies and proportions. A student's *t* test was used for comparisons between two groups, and one-way analysis of variance (ANOVA) test was used to determine the statistical significance of multiple groups. A nonparametric test or a *χ*^2^ test was used where appropriate to estimate the differences between groups. We calculated the odds ratios (ORs) and 95% confidence intervals (CIs) by using logistic regression models. The area under the receiver operator characteristic (ROC) curves was calculated as a measure of discriminative ability for finding the accuracy of serum chymase and tryptase levels in predicting fatal SFTS. All analyses were performed using Stata 14.0 (Stata Corp. LP, College Station, TX, USA). A two-sided *P* < 0.05 was considered statistically significant.

### Data availability.

The study design, protocol, and statistical analysis are provided in the main manuscript and the supplementary data files. Access to the data generated and analyzed in this study will be provided upon reasonable request to the corresponding author.
