# Supplementary material for: Mast Cell-Derived Proteases Induce Endothelial Permeability and Vascular Damage in Severe Fever with Thrombocytopenia Syndrome
Source: Microbiol Spectr. 2022 May 25;10(3):e01294-22. doi: 10.1128/spectrum.01294-22 (PMC9241724; doi:10.1128/spectrum.01294-22)
Supplement: SUPPLEMENTAL FILE 1 — Supplemental material. Download spectrum.01294-22-s001.pdf, PDF file, 0.3 MB [file spectrum.01294-22-s001.pdf]

## Supplementary methods

### β-hexosaminidase release assay

A standard β-hexosaminidase assay was used to assessed *in vitro* MCs degranulation. Briefly, LAD2 cells were exposed to SFTSV at various multiplicity of infection (MOI) for 1 hour. Cell suspension was collected and separated by centrifugation after the incubation period, and the cell pellet was lysed by 1% Triton X-100. β-hexosaminidase levels were measured in both the supernatant and cell lysate and detected by p-nitrophenyl-N-acetyl-β-D-glucosaminide (Sigma, USA) in 0.09 M citrate buffer (Sigma), followed by the addition of 0.1 M sodium carbonate buffer (Sigma) to terminate the reaction. Absorbance was recorded at 405nm with an automated microplate reader (Multiskan GO, USA). The net percentage of β-hexosaminidase release was calculated to estimate percent degranulation as following formula: %Degranulation = [(β-hexosaminidase in supernatant) / (β-hexosaminidase in supernatant + β-hexosaminidase in pellet)] × 100%.

### Transwell assay

The transendothelial electric resistance (TER) readings of HUVEC cells were measured using the Millipore Millicell-ERS (Electrical Resistance System). Briefly, HUVEC cells were grown on upper chamber of transwell inserts (Corning, USA) until form a monolayer. MCs were treated with either SFTSV or mock media (untreated). Cell debris was removed, followed by further centrifugation at 12,000 g for 10 min to obtained soluble fractions from SFTSV-induced MC total media and cellular precipitate was resuspended in maintenance media. SFTSV-induced MC total media, soluble and

particulate fractions were exposed to HUVEC monolayers. At the same time, the transwell inserts without HUVEC cells was measured as the baseline. Monolayer permeability was measured by acquiring TER readings. The normalized TER was calculated via the following formula: TER (% of initial value) = experimental TER value/baseline TER value ×100%.

**Immunofluorescence assay**

The treated cells were fixed with paraformaldehyde, permeabilized with Triton-100, and blocked using BSA blocking buffer. Cells were stained with mouse polyclonal antibody against SFTSV NP or rabbit polyclonal antibody against ZO-1 (Invitrogen, USA), followed by addition of the secondary FITC Conjugate Goat Anti-Mouse IgG (Transgen, China) or Alexa Fluor 488 donkey anti-rabbit IgG (Invitrogen, USA), and counterstained with a drop of ProLongTM Gold Antifade reagent containing DAPI (Invitrogen, USA). Cell staining images were obtained with the Leica DMi8 system (Leica, German).

**Toluidine blue staining**

Severe fever with thrombocytopenia syndrome virus (SFTSV) or normal saline was injected into mouse footpads. The paraffin-embedded footpad tissue was cut in sections, and fixed with Carnoy's fixative, followed by staining with 0.1% solution of toluidine blue.

**Flow cytometry**

The mouse footpads were challenged with chymase and tryptase or SFTSV, and digested into single-cell suspensions using collagenase (Merk, USA), followed by

filtering the cell strainer (BD Biosciences, USA). Cells were collected by centrifugation, stained with FITC anti-mouse CD31 antibody (Biolegend, USA) and PE/Cyanine7 anti-mouse CD45 antibody (Biolegend, USA). The acquired data were analyzed by NovoCyte Flow Cytometer (ACEA Biosciences, USA) with NovoExpress® software.

**Quantitation of viral load**

The total RNA was extracted using the QIAamp MinElute Virus Spin Kit (Qiagen, German). Virus loads were determined through qRT-PCR. The primers and probe used were as follows: 5′- TTCACAGCAGCATGGAGAGG -3′ (forward primer), 5′-GATGCCTTCACCAAGACTATCAATG -3′ (reverse primer), FAM 5′-AACTTCTGTCTTGCTGGCTCCGC-BHQ1-3′ (probe) (1). The qRT-PCR was performed with the One-step Primer Script RT-PCR Kit (Takara, Japan) in a LightCycler 480 (Roche). Plasmids containing known copy number of amplification targets were included in PCR assays to generate a standard curve for quantification of test samples. The copy number of SFTSV was determined by comparison with a serially diluted plasmid standard of known concentration. All samples were quantified in at least duplicate wells. Positive and negative controls were included in all assays.

**Human clinical samples**

Samples were retrospectively obtained from confirmed severe fever with thrombocytopenia syndrome (SFTS) patients in the PLA 990 hospital, Xinyang in Henan province. Totally 147 patients enrolled in the study. All patients were laboratory-confirmed case of SFTS. The recruited cases had their serum samples collected at the acute phase of illness when admitted to the hospital and at the convalescent phase when

discharged from the hospital. For the deceased patients, the second samples were collected at the time of disease deterioration. Healthy blood donors (n = 12) from the physical examination department of the same hospital with comparable age and gender, who were both SFTSV negative by qRT-PCR and serological tests were selected as the control group. Informed consent was obtained from all individuals, and the study was performed with approval from the Ethical Committee of Beijing Institute of Microbiology and Epidemiology.

**Figure S1. Comparison of PECAM-1, VEGF-A, SAA-1, VCAM-1, ICAM-1, P-selectin, E-selectin, tPA and PAI-1 stratified by level of serum chymase and tryptase content.**

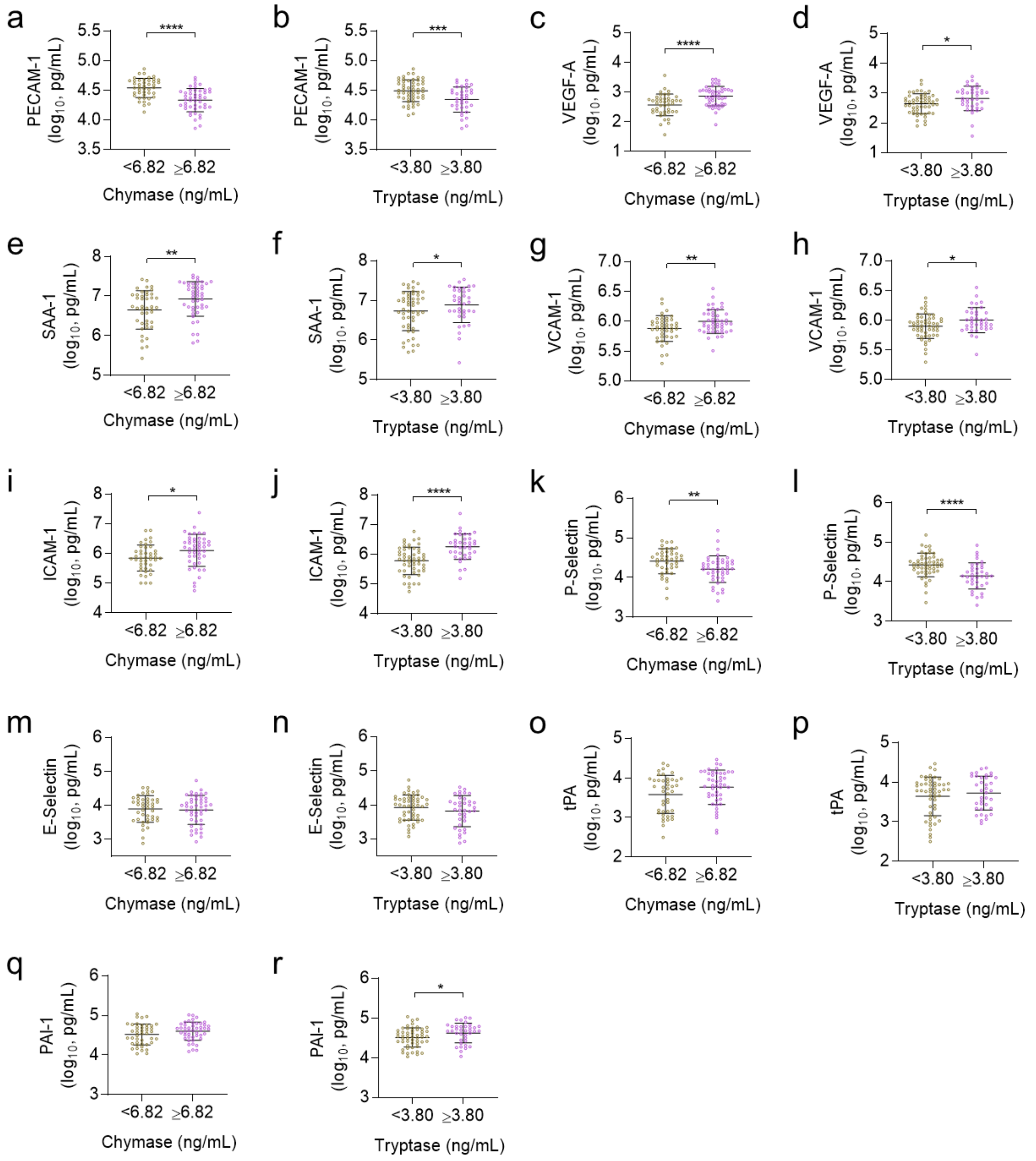


The groups were classified by median values (n = 43 for chymase < 6.82 ng/mL; n = 46 for chymase ⩾ 6.82 ng/mL; n = 52 for tryptase < 3.80 ng/mL; n = 37 for tryptase ⩾ 3.80 ng/mL). Student's *t* test was performed to determine significance between groups. **P* < 0.05, ***P* < 0.01, ****P* < 0.001, *****P* < 0.0001. Dots indicate exact value; horizontal lines indicate mean and SD.

**Table S1. The basic characteristics and clinical manifestations of SFTS cases and healthy controls.**

| Characteristics | SFTS cases (n = 147) | | | Controls (n = 12) | *P* value |
|---|---|---|---|---|---|
| | Total | Surviving (n = 108) | Deceased (n = 39) | | |
| **Epidemiological characteristics** | | | | | |
| Female gender, No. (%) | 91 (61.9) | 69 (63.9) | 22 (56.4) | 6 (50.0) | 0.613 |
| Age, years, median (IQR) | 66 (60, 71) | 64. (58, 71) | 69 (63, 73) | 65 (65, 70) | 0.523 |
| Hospitalized day, days, median (IQR) | 8 (5.5-11) | 10 (8-12) | 4 (3-5) | | |
| Interval from onset to admission, days, median (IQR) | 5 (4-6) | 5 (4-6) | 5 (3.5-7) | | |
| **Clinical manifestation** | | | | | |
| **Bleeding symptom, No. (%)** | | | | | |
| Melena | 12 (8.2) | 8 (7.4) | 4 (10.3) | | |
| Gingival bleeding | 26 (17.7) | 22 (20.4) | 4 (10.3) | | |
| Hemoptysis | 6 (4.1) | 2 (1.9) | 4 (10.3) | | |
| Hematemesis | 2 (1.4) | 1 (0.9) | 1 (2.6) | | |
| Petechiae | 5 (3.4) | 5 (4.6) | 0 (0.0) | | |
| Ecchymosis | 30 (20.4) | 24 (22.2) | 6 (15.4) | | |
| Epistaxis | 3 (2.0) | 2 (1.9) | 1 (2.6) | | |
| Oral Hemorrhage | 4 (2.7) | 3 (2.8) | 1 (2.6) | | |
| Hematuria | 7 (4.8) | 6 (5.6) | 1 (2.6) | | |
| **Vascular leakage symptom, No. (%)** | | | | | |
| Hydrothorax | 23 (15.6) | 17 (15.7) | 6 (15.4) | | |
| Hydropericardium | 5 (3.4) | 4 (3.7) | 1 (2.6) | | |
| Pelvic effusion | 2 (1.4) | 1 (0.9) | 1 (2.6) | | |
| Ascites | 3 (2.0) | 3 (2.8) | 0 (0.0) | | |
| Hydropsia | 8 (5.4) | 7 (6.5) | 1 (2.6) | | |
| **Neurological symptoms, No. (%)** | | | | | |
| Apathy | 6 (4.1) | 6 (5.6) | 0 (0.0) | | |
| Dysphoric | 37 (25.2) | 32 (29.6) | 5 (12.8) | | |
| Convulsion | 29 (20.0) | 22 (20.6) | 7 (18.4) | | |
| Somnolence | 11 (7.5) | 9 (8.3) | 2 (5.1) | | |
| Blurred mind | 37 (25.2) | 28 (25.9) | 9 (23.1) | | |
| Lethargy | 5 (3.4) | 3 (2.8) | 2 (5.1) | | |
| Coma | 15 (10.2) | 13 (12.0) | 2 (5.1) | | |
| Trembling | 19 (12.9) | 14 (13.0) | 5 (12.8) | | |
| **Comorbidities, No. (%)** | | | | | |
| Any comorbidity | 45 (30.6) | 27 (25.0) | 18 (46.2) | | |
| Hypertension | 17 (11.6) | 12 (11.1) | 5 (12.8) | | |

| | | | |
|---|---|---|---|
| Chronic viral hepatitis | 16 (10.9) | 12 (11.1) | 4 (10.3) |
| Diabetes | 12 (8.2) | 5 (4.6) | 7 (17.9) |
| Chronic obstructive pulmonary disease | 6 (4.1) | 3 (2.8) | 3 (7.7) |
| Coronary heart disease | 2 (1.4) | 1 (0.9) | 1 (2.6) |

*P* values (SFTS cases v.s Controls) were calculated by a non-parametric test, or a $\chi^2$ test.

**Table S2. Multivariate logistic regression analysis of serum MC-specific proteases chymase and tryptase in the acute phase associated with fatal outcome by adjusting age, sex and delay from symptom onset to hospital admission.**

| | Total (n = 147) | Fatal patients (n = 39) | Surviving patients (n = 108) | Adjusted OR (95% CI) | *P* value |
|---|---|---|---|---|---|
| **Chymase** | | | | | |
| < 6.82 ng/mL | 74 | 12 (30.77) | 62 (57.41) | 3.01 (1.35, 7.11) | 0.009 |
| ≥ 6.82 ng/mL | 73 | 27 (69.23) | 46 (42.59) | | |
| **Tryptase** | | | | | |
| < 3.80 ng/mL | 74 | 14 (35.90) | 60 (55.56) | 2.23 (1.01, 5.10) | 0.05 |
| ≥ 3.80 ng/mL | 73 | 25 (64.10) | 48 (44.44) | | |

The levels of chymase and tryptase were divided by median values.

*P* values were calculated by multivariate logistic regression model.

## Reference


1. Lu QB, Li H, Zhang PH, Cui N, Yang ZD, Fan YD, Cui XM, Hu JG, Guo CT, Zhang XA, Liu W, Cao WC. 2016. Severe Fever with Thrombocytopenia Syndrome Complicated by Co-infection with Spotted Fever Group Rickettsiae, China. Emerg Infect Dis 22:1957-1960.
